# A Permeable Cuticle, Not Open Stomata, Is the Primary Source of Water Loss From Expanding Leaves

**DOI:** 10.3389/fpls.2020.00774

**Published:** 2020-06-23

**Authors:** Cade N. Kane, Gregory J. Jordan, Steven Jansen, Scott A. M. McAdam

**Affiliations:** ^1^ Department of Botany and Plant Pathology, Purdue University, West Lafayette, IN, United States; ^2^ School of Natural Sciences, University of Tasmania, Hobart, TAS, Australia; ^3^ Institute of Systematic Botany and Ecology, Ulm University, Ulm, Germany

**Keywords:** plant cuticle, Quercus-oak, leaf development, abscisic acid, stomatal development, stomata, plant physiology, cuticle development

## Abstract

High rates of water loss in young, expanding leaves have previously been attributed to open stomata that only develop a capacity to close once exposed to low humidity and high abscisic acid (ABA) levels. To test this model, we quantified water loss through stomata and cuticle in expanding leaves of *Quercus rubra*. Stomatal anatomy and density were observed using scanning electron microscopy. Leaves of *Q. rubra* less than 5 days after emergence have no stomata; therefore, water loss from these leaves must be through the cuticle. Once stomata develop, they are initially covered in a cuticle and have no outer cuticular ledge, implying that the majority of water lost from leaves in this phase of expansion is through the cuticle. Foliar ABA levels are high when leaves first expand and decline exponentially as leaves expand. Once leaves have expanded to maximum size, ABA levels are at a minimum, an outer cuticular ledge has formed on most stomata, cuticular conductance has declined, and most water loss is through the stomata. Similar sequences of events leading to stomatal regulation of water loss in expanding leaves may be general across angiosperms.

## Introduction

Expanding leaves are highly sensitive to abiotic stresses including drought stress (Hsiao and Xu, 2000; Pantin et al., 2012). Yet, somewhat paradoxically, there are reports of extremely high rates of evaporation from young, expanding leaves (Pantin et al., 2013). High rates of water loss in young leaves have been attributed to open stomata that are unable to close because they lack sensitivity to abscisic acid (ABA) (Pantin et al., 2013). A major assumption in this model is that the physical characteristics of expanding leaves are similar to those of fully developed leaves. However, several factors challenge this assumption. Cell turgor dynamics are different between expanding and fully developed leaves, with expanding leaves maintaining high cell turgor essential for both cell expansion and the supply of nutrients to developing tissues (Shackel et al., 1987; Hsiao and Xu, 2000; Liu et al., 2003; Siebrecht et al., 2003; Sansberro et al., 2004). Cell walls in expanding leaves must be highly flexible to allow for cell expansion (Schultz and Matthews, 1993), but normal stomatal function requires rigid cell walls (Buckley et al., 2003). In addition, the cuticle, a waxy layer that forms on the outer wall of the epidermal cells of all terrestrial plants (Raven, 1984; Gülz, 1994; Schreiber and Riederer, 1996), has been dismissed as a major source of water loss in expanding leaves (Pantin et al., 2013). This is despite reports that cuticular conductance can be very high in young leaves and decreases during leaf expansion (Hamerlynck and Knapp, 1996; Hauke and Schreiber, 1998). These ontogenetic changes may reflect changes in the cuticle during leaf expansion: during the initial phase of rapid epidermal cell expansion the cuticle remains thin, elastic, and often disjointed with epidermal cell-shaped pieces of cuticle sitting on top of epidermal cells (Sargent, 1976). Once leaf expansion ceases, the cuticle thickens, completely covering the leaf surface, while becoming firm and rigid (Sargent, 1976; Onoda et al., 2012).

The evolution of the cuticle is believed to have allowed the aquatic algal ancestors of land plants to colonize terrestrial environments (Raven, 1984; Edwards et al., 1996; Kenrick and Crane, 1997). Despite being present on all terrestrial plants, the cuticle can vary markedly in thickness, composition, and conductance at the interspecific level, and across various developmental stages and organs within an individual plant (Jeffree, 1996; Goodwin and Jenks, 2005; Buschhaus et al., 2007; Fernández et al., 2016). Being predominantly hydrophobic wax, fully developed cuticles provide a near-water tight seal on the outside of cell walls, protecting internal tissues from desiccation, blocking UV light, and acting as barrier against pathogens and physical abrasion (Edwards et al., 1996; Krauss et al., 1997; Łaźniewska et al., 2012).

Recent work suggests that cuticular organic compounds are formed within epidermal cells and transported to the outside of the cell wall *via* transport proteins, after which the cuticle self-assembles by evaporation (Lee and Priestley, 1924; Neinhuis et al., 2001; Schreiber, 2005; Yeats and Rose, 2013). While cuticles are deposited by evaporation, they also create an almost gas-tight seal around the cells (Lendzian, 1982; Lendzian and Kerstiens, 1991). The low permeability to gases severely limits CO_2_ diffusion, which provided a strong selective pressure for the evolution of stomata, the epidermal valves that provide internal photosynthetic cells with access to atmospheric CO_2_ (Lendzian, 1982; Lendzian and Kerstiens, 1991; Brodribb et al., 2020).

A waterproof cuticle punctuated with stomatal valves to facilitate gas exchange is essential for homoiohydry and plant growth in the desiccating environments that almost all vascular plants occupy (Lendzian, 1982; Raven, 1984; Brodribb et al., 2020). In a hydrated plant, stomata account for more than 99% of total water loss from a leaf, but once stomata close during a drought, it is believed that a considerable proportion of water lost from the plant evaporates *via* the cuticle (Körner, 1993; Duursma et al., 2019). After drought-induced closure of stomata, between 50 and 94% of the water lost from leaves is reported to be lost through the cuticle or incompletely closed stomata (Šantrůček et al., 2004; Brodribb et al., 2014). Much like the variation in maximum stomatal conductance (Körner et al., 1979), the degree of variation in cuticular conductance between species can be considerable and may be critical for determining the ecological limits of species (Schreiber and Riederer, 1996; Mayr, 2007). Highly permeable cuticles are found in moss and fern gametophytes, while very low cuticular conductance is found in species that are adapted to dry environments (Edwards et al., 1996; Jeffree, 1996; Schreiber and Riederer, 1996; Brodribb et al., 2014; Blackman et al., 2016; Carignato et al., 2020; Lee et al., 2019). Pollutants and time can degrade the leaf cuticle impacting drought resistance (Jordan and Brodribb, 2007; Burkhardt and Pariyar, 2014). In particular, the removal of outer cuticular waxes can severely decrease drought tolerance in semiarid woody species, leading to a reduction in photosynthesis, gas exchange, and plant pigment levels (Medeiros et al., 2017; Pereira et al., 2019).

Although there has long been a focus on cuticular conductance in determining drought-tolerance thresholds, almost no focus has been placed on the role of cuticular conductance in determining leaf gas exchange as leaves expand. Complete leaf expansion in *Hedera helix* occurs around the same time cuticular conductance reaches a minimum (Hauke and Schreiber, 1998). Cuticles also appear to cease developing in chemical composition once leaves cease expanding (Hauke and Schreiber, 1998). Furthermore, very young stomata are covered in a cuticle (Davis and Gunning, 1993; Nadeau and Sack, 2002; Hunt et al., 2017). Breaking of this cuticle covering layer in leaf development to form the outer cuticular ledge may be responsible for reported increases in leaf gas exchange as leaves expand (Constable and Rawson, 1980). In support of this rates of gas exchange in mutant plants of *Arabidopsis* in which stomata are occluded by a cuticle covering are half that of wild-type plants without occluded stomata (Hunt et al., 2017).

Here, we utilize the hypostomatic species *Quercus rubra* to separate cuticular and stomatal water loss from total leaf transpiration in expanding leaves. *Q. rubra* has large, fast-growing leaves, making it ideal for these experiments. We reexamine the ontogeny of the formation of the outer cuticular ledge in expanding *Arabidopsis* leaves, which is essential for the initiation of stomatal conductance. We also collected foliage ABA levels in expanding leaves to examine what, if any, role ABA may play in “priming” stomatal function.

## Materials and Methods

### Plant Material

Six, 3 year-old bare-rooted *Q. rubra* plants were planted in 10 L pots containing a 1:1:1 mix of Indiana Miami topsoil, ground pine bark, and sand. Plants were grown in the glasshouses of Purdue University, IN, USA, under a 16 h photoperiod, supplemented, and extended with LED lights (Illumitex Power Harvest I4, TX, USA) that provided a photon flux density on an F3 spectrum (22.4% blue; 13.4% green; 63.9% red; and 0.4% far-red) of 150μmol m^−2^ s^−1^ at pot level. The highest PPFD (natural and supplemental light) measured was 1,800μmol m^−2^ s^−1^ at solar noon on a cloudless day. Plants were watered daily and received liquid nutrients once per month. Conditions in the glasshouse were set at a night/day temperature of 22/28°C. After initial bud burst, all developing leaves were tagged with the date of leaf emergence. Six plants of *Arabidopsis thaliana Col-0* were grown under a 10 h photoperiod, supplied by LED lights (SUNCO Lighting, CA, USA), providing a photon flux density of 60 μmol m^−2^ s^−1^ at pot level. Seeds were sown directly on germination mix (Sun Gro Horticulture, MA, USA). Plants were watered from the base and given liquid nutrients once per month. Plants were imaged daily to determine leaf age. The area of eight leaves was measured daily from initial emergence until 23 days after emergence.

### Determining Cuticular and Stomatal Conductance by Leaf Gas Exchange

Leaf gas exchange was measured using an infrared gas analyzer (LI-6800, Licor Biosciences, NE, USA). Conditions in the leaf cuvette were maintained as close to ambient glasshouse conditions as possible, and light conditions were set at 1,500 μmol m^−2^ s^−1^. Measurements were taken between 09:00 till 11:00 on clear, cloudless days. Initial stomatal conductance (*g_s_*) was measured on expanding, or fully expanded, leaves by enclosing the leaf in the chamber and measuring instantaneous leaf gas exchange parameters. After this initial measurement, the abaxial surface of the leaf was covered in petroleum jelly and plastic wrap and instantaneous leaf gas exchange was again measured in the same region of the leaf, or the whole leaf. By covering the abaxial leaf surface we only measured gas exchange through the adaxial surface which has no stomata or hydathodes, like most *Quercus* species (Bolhàr-Nordenkampf and Draxler, 1993; Ivănescu et al., 2009). All rates of leaf gas exchange were normalized by leaf area in the cuvette. Whole leaf area was also measured for each leaf analyzed by imaging leaves (12 megapixel, IPhone 7, Apple Inc., CA, USA) and measuring area using ImageJ (National 303 Institutes of Health, Bethesda, MD, USA). To avoid variation due to potential developmental variation across the leaf surface, the center of each leaf was placed in the cuvette. In younger leaves, we were able to measure the whole leaf. All measured leaves were preserved in methanol and stored at −20°C for anatomical assessment. Cuticular and stomatal conductance and the percent of total leaf conductance that occurred through the stomata were calculated according to Jordan and Brodribb (2007).

### Quantifying Foliage Abscisic Acid Levels

Leaves were harvested at 11:00 and immediately wrapped in damp paper towel and bagged. A sample of tissue was taken from each leaf, weighed (±0.0001 g, OHAUS Corporation, NJ, USA) and then covered in −20°C 80% methanol in water (v v^−1^) containing 250 mg L^−1^ butylated hydroxytoluene, chopped to fine pieces and stored at −20°C overnight. Extraction in methanol ensures that both free and fettered ABA in the chloroplasts were extracted from the sample (Georgopoulou and Milborrow, 2012). The samples were homogenized and 15 μl of deuterium labeled [^2^H_6_]ABA (OlChemim Ltd, Czech Republic) was added as an internal standard. ABA was extracted overnight at 4°C. An aliquot of supernatant was dried in a vacuum sample concentrator (Labconco, MO, USA), and ABA was resuspended in 200 μl of 2% acetic acid in water (v v^−1^), centrifuged at 14,800 RPM for 4 min and 100 μl taken for analysis. The level of ABA and internal standard in each sample was quantified using an Agilent 6460 series triple quadrupole LC/MS (Agilent, CA, USA) according to McAdam (2015). After quantification, the plant material from which the supernatant was taken was dried down at 70°C, and leaf dry weight was estimated by subtracting the initial mass of the empty tube.

### Anatomy

Stomatal anatomy was analyzed in hole punches (diameter 0.5 cm) from the center of *Q. rubra* leaves ranging from 1 to 30 days of age (including all of the leaves measured for leaf exchange) that had been stored in methanol at −20°C. Anatomical samples were collected from either the whole leaf, in young leaves or from center of the leaves when they were large enough. In *Q. rubra*, leaves expand evenly and then acropetally after reaching approximately 70% of maximum size (Tomlinson et al., 1991); our sampling protocol ensured that we avoided these regions of differential or continual expansion in larger leaves. Samples were prepared for SEM by critical point drying (E3000 Critical Point Dryer, Quorum Technologies, East Sussex, UK). Dried samples were placed on stubs and sputter coated for 60 s at 8 mA using a gold target (Balzers Union FL-9496 sputter device, Balzers, Liechtenstein). Images of stomata from the abaxial surface were taken on a Phenom XL desktop SEM (Nano Science Instruments, AZ, USA) at 1,000x magnification to determine stomatal density and the percent of stomata in which the outer cuticular ledge had formed. For stomatal density measurements, a stoma was counted if both guard cells were discernible. A stoma with an outer cuticular ledge was defined as having any form of rip, tear, or hole in the cuticular covering over the stomatal pore. Cross sections of *Q. rubra* leaves were made using a freezing microtome (Microm HM 430, Thermo Scientific, MA, USA). The cuticle on leaf sections was stained using Sudan IV (0.5 g powdered Sudan IV in 100 ml 75% Ethanol, 25% DI water) for 8 h at 25°C. Images were taken using a 40x oil emersion objective on a light microscope (AxioImagerA2, Zeiss, Germany). Observations were made from four different sections from three different leaves 6 and 21 days after emerging.

*Arabidopsis* leaves used for stomatal anatomy were harvested on a single day and stored in methanol at −20°C. Leaf segments were prepared to observe the abaxial leaf surface and attached to a SEM stub with 1:1 OCT Cryo-Gel and water. Leaf pieces were frozen in a liquid nitrogen slurry and moved into a Gatan Alto 2500 (Gatan 316 Inc., Pleasanton, CA, USA) cryo-preparation chamber of an SEM (FEI Nova Nano 317200, Hillsboro, OR, USA). The samples were placed under vacuum and held at −170°C. Samples were then allowed to sublimate at −90°C, while viewing to remove frost. Leaves were sputter coated for 120 s at 8 mA using a platinum target and then imaged at −140°C.

### Leaf Water Potential

Midday leaf water potential was measured in young expanding leaves (6 days after leaf emergence), as well as fully expanded leaves (32 days after leaf emergence) using a Scholander pressure chamber (PMS Instrument Company, OR, USA). Leaves were excised and wrapped in damp paper towel and immediately placed into a humid plastic bag. Leaves were allowed to equilibrate in dark, in the humid bag for 5 min before measurements were taken.

## Results

**Figure 1:**
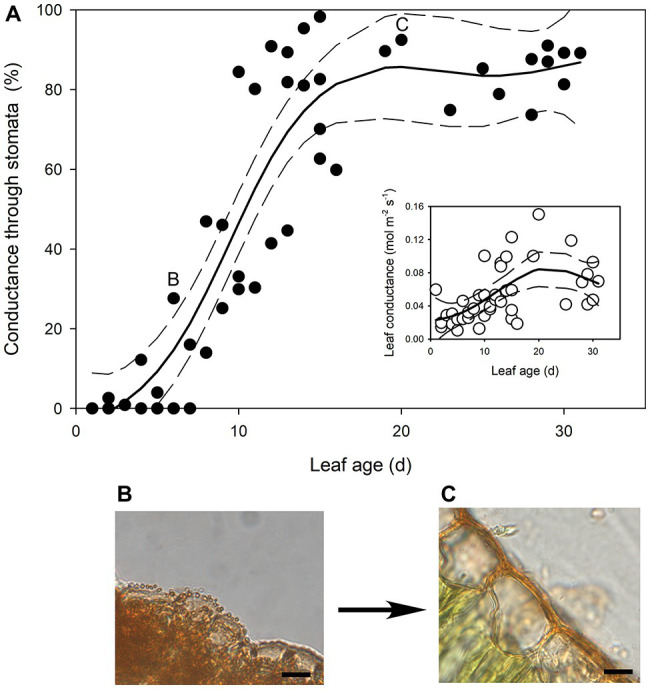
**(A)** The percentage of transpired water lost through stomata as *Quercus rubra* leaves expand. The insert depicts the absolute rates of leaf conductance measured in the same leaves. Generalized additive model curves and 95% confidence intervals are represented by solid and dashed black lines, respectively. Each point represents a single leaf. Letters on the chart depict the leaf from which representative images (**B**,**C**) were taken. (**B**) Cross sections through the epidermis of a *Q. rubra* leaf 6 days after emerging, and (**C**) 21 days after emerging, with cuticles stained using Sudan IV (scale bars = 10 μm).

**Figure 2:**
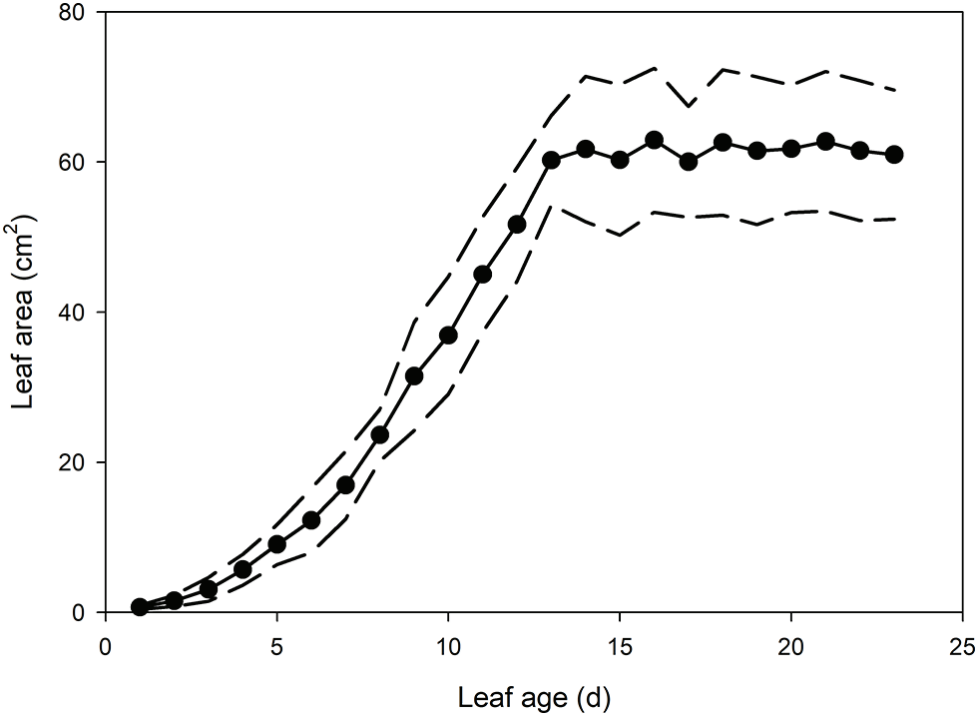
Mean leaf area of *Q. rubra* leaves from emergence (day 0) to 23 days after leaf emergence (*n* = 8 leaves, ± *SD*). Dashed lines depict standard deviation.

In the newest expanding leaves of *Q. rubra* (less than 5 days old; i.e., at ~15% of fully expanded area), whole leaf conductance was found to be relatively high, at 0.023 mol m^−2^ s^−1^. By 10 days after leaf emergence (i.e., at 60% of fully expanded area), leaf conductance had doubled to 0.047 mol m^−2^ s^−1^ (Figures 1, 2). While leaf conductance was measurable in leaves that were less than 5 days old, less than 5% of total leaf conductance was found to be lost through the stomata (Figure 1). After 5 days of leaf expansion, the percentage of water lost from a leaf through stomata began to increase rapidly (Figure 1). Ten days after leaf emergence, the stomata were found to be responsible for approximately 50% of water loss from the leaf (Figure 1). By 15 days after leaf emergence, the percentage of water lost through the stomata accounted for more than 80% of total leaf conductance, which had increased to more than 0.075 mol m^−2^ s^−1^ (Figure 1). By this age leaves were fully expanded. In general, leaves had ceased to expand by day 13 (Figure 2). Leaves 6 days after emerging did not appear to have a very thick or well-developed cuticle when compared to leaves 21 days after emerging, which displayed a much thicker and well-developed cuticle (Figure 1).

**Figure 3:**
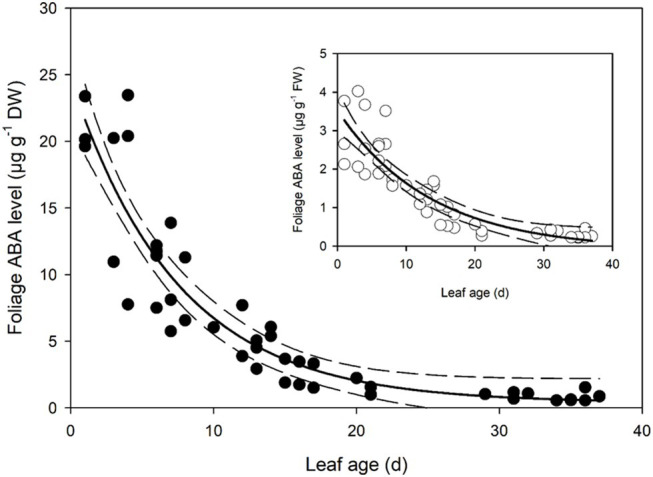
Foliage abscisic acid (ABA) level in expanding *Q. rubra* leaves. ABA levels are expressed in terms of dry weight. A single exponential decay three parameter model (ABA level *DW* = 0.3822 + 24.2829 × e^−0.1340 × Leaf age^) (solid line) with 95% confidence interval (dashed lines) is depicted (*p* = <0.0001, *R* ^2^ = 0.8493). The insert shows ABA levels in terms of fresh weight (FW). A single exponential decay three parameter model (ABA level *FW* = −0.0982 + 3.6244 × e^−0.0737 × Leaf age^) (solid line) with 95% confidence interval (dashed line) is depicted (*p* = <0.0001, *R* ^2^ = 0.7912).

Foliar ABA levels in developing *Q. rubra* leaves were approximately 21.5 μg g^−1^ dry weight on the first day following leaf emergence (Figure 3). As leaves expanded, this high level of initial ABA in primordial leaves declined following an exponential decay curve, such that by 7 days after leaf emergence, ABA levels in terms of dry weight were half the initial level in the newest emerged leaves (Figure 3). ABA levels continued to decline until around 30 days after initial leaf emergence, by which time they had approached a steady-state level of around 0.55 μg g^−1^ dry weight (Figure 3).

**Figure 4:**
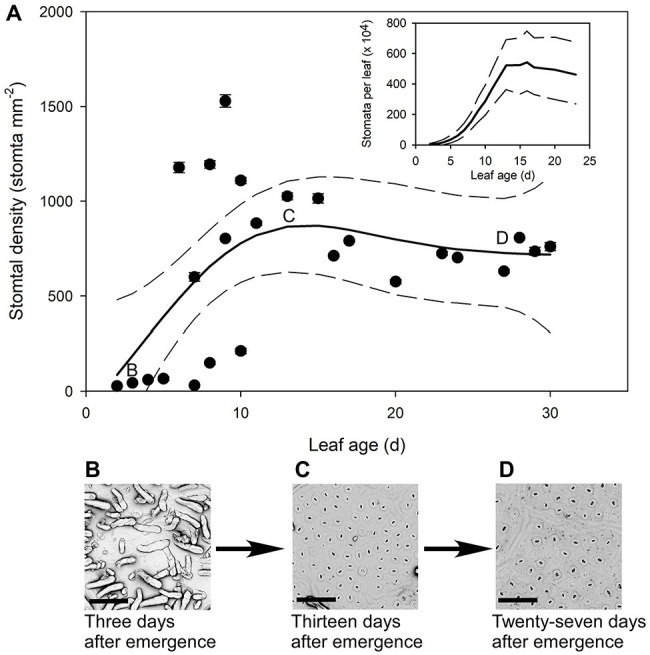
(**A**) Mean stomatal density (*n* = 5 fields of view per leaf taken from the center of the leaf, ± *SE*) of expanding *Q. rubra* leaves. Each point represents a single leaf. Letters on the chart depict the leaf from which representative images (**B**–**D**) were taken. Generalized additive model curves and 95% confidence intervals are represented by solid and dashed black line respectively. The insert represents the total number of stomata per leaf of expanding *Q.rubra* leaves (solid line) flanked by the 95% confidence interval (dashed line). (**B**) An image of the abaxial surface of a *Q. rubra* leaf 3 days after emergence with visible trichomes (scale bar = 80 μm). (**C**) An image of the abaxial surface of a *Q. rubra* leaf 13 days after emergence (scale bar = 80 μm). (**D**) An image of the abaxial surface of a *Q. rubra* leaf 27 days after emergence (scale bar = 80 μm).

The youngest *Q. rubra* leaves had very few stomata, with approximately 27 ± 2 stomata mm^−2^ by the second day following emergence (Figure 4). Stomatal densities remained low in expanding leaves until 5 days after leaf emergence, when densities rapidly increased by 20-fold, to approximately 575 stomata mm^−2^ (Figure 4). Allowing for a change in leaf area, this indicates a 200,000-fold increase in the total number of stomata over that time (Figure 4). The highest recorded stomatal density on an individual leaf was measured in leaves 9 days after leaf emergence, with 1,528 ± 33 stomata mm^−2^ (Figure 4), after which stomatal density declined as leaves continued to expand. Seventeen days after leaf emergence, stomatal density reached a steady-state mean density of 790 stomata mm^−2^ (±5) (Figure 4).

**Figure 5:**
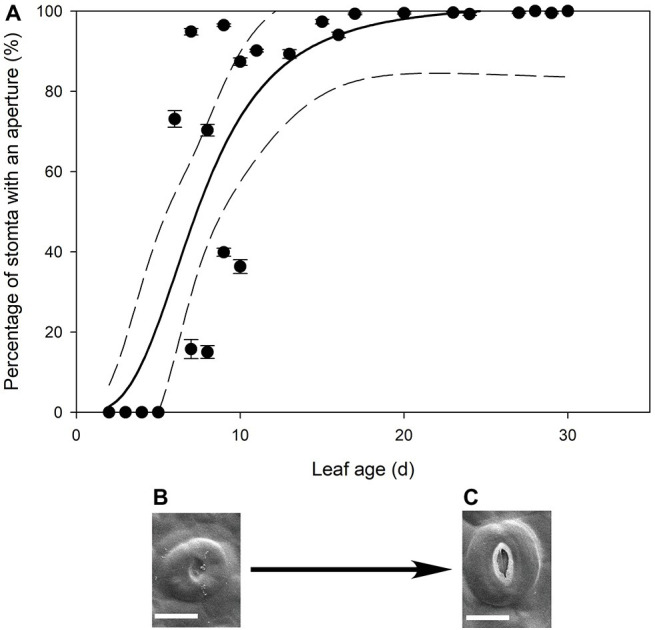
(**A**) Mean percentage of stomata with an aperture (*n* = 5 fields of view per leaf taken from the center of the leaf, ± *SE*) in expanding leaves of *Q. rubra*. Each point represents a single leaf. A logistic three parameter sigmoidal curve (solid line) and 95% confidence interval (dashed line) is shown (*p* = <0.0001, *R* ^2^ = 0.7178). (**B**,**C**) Representative images of *Q. rubra* stomata (**B**) without an aperture and (**C**) with an aperture captured on the same leaf 10 days after emergence (scale bar = 10 μm).

**Figure 6:**
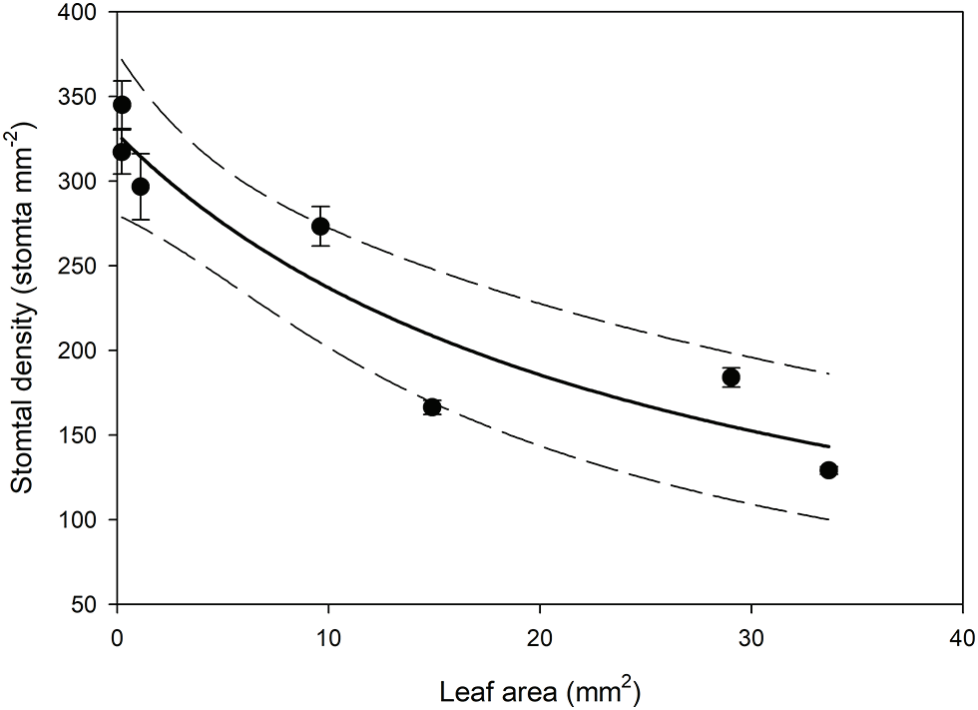
Mean stomatal density on the abaxial surface (*n* = 5 fields of view from the same leaf taken from the center of the leaf, ± *SE*) in expanding *Arabidopsis thaliana Col-0* leaves. Each point represents a single leaf. A rational, 2 Parameter II curve (solid line) and 95% confidence interval (dashed line) is shown (*p* = <0.0015, *R* ^2^ = 0.8870).

**Figure 7:**
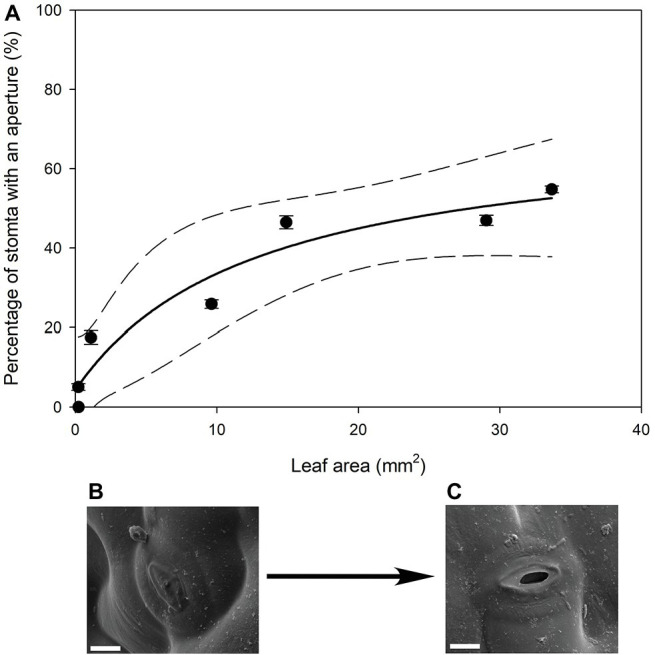
(**A**) Mean percentage of stomata that have formed an aperture on the abaxial surface (*n* = 5 fields of view per leaf taken from the center of the leaf, ± *SE*) in young expanding leaves of *A. thaliana Col-0*. Each point represents a single leaf. A rational, 3 Parameter II (solid line) and 95% confidence interval (dashed line) is shown (*p* = <0.0050, *R* ^2^ = 0.9295). (**B**) Image of an *A. thaliana Col-0* stoma without an aperture on a leaf that was 29.04 mm^2^, approximately 6 days after emergence (Scale bar = 5 μm). (**C**) Image of an *A. thaliana Col-0* stoma with an aperture on with the same leaf imaged in (**B**) (Scale bar = 5 μm).

In all stomatal complexes on leaves younger than 7 days old, a cuticle covered the pore between the guard cells (Figure 5). The presence of this covering meant that these stomatal complexes did not have apertures and therefore could not be functional stomata. By 13 days after leaf emergence, in 90% of stomatal complexes, this cuticle layer had split to create an aperture and an outer cuticular ledge (Figure 5). Similar patterns in the formation of the outer cuticular ledge were observed in the expanding leaves of *A. thaliana Col-0* plants (Figures 6, 7) with most stomata in the smallest and youngest leaves covered with cuticle (Figure 7). Zero to five percent of stomata had formed an outer cuticular ledge in leaves of *A. thaliana* that were <0.25 mm^2^ in area and had not yet emerged from the center of the rosette. Once leaves had emerged from the rosette for approximately 1 day (being more than 10 mm^2^ in area), approximately 25% of the stomata had developed an outer cuticular ledge (Figure 7). The number of stomata forming an outer cuticular ledge per day declined once *A. thaliana* leaves reached approximately 15 mm^2^ in area.

We found that leaf water potential of young expanding leaves of *Q. rubra* was the same as that of fully expanded leaves on the same plant. Leaves 3 days after emerging had a water potential of −0.866 ± 0.113 MPa (*n* = 3, *SE*), while leaf water potential in leaves that emerged at least 32 days prior to the measurement, and were fully expanded, was −0.763 ± 0.089 MPa (*n* = 3, *SE*).

## Discussion

Contrary to the model of Pantin et al. (2013), based on observations in *Arabidopsis*, cuticular conductance accounts for the majority of water loss from expanding leaves in *Q. rubra*. In *Q. rubra* the youngest leaves have no stomata and once stomata form, they have no aperture as they are still covered in cuticle. Only once the stoma and aperture forms by tearing the covering cuticle do stomata become the primary source of leaf conductance to water vapor. We found no evidence in *Q. rubra* that ABA levels increased as leaves expand, thereby priming stomata to function as hypothesized by Pantin et al. (2013). In contrast, ABA levels were very high in young expanding leaves and appeared to decline thereby, presumably, allowing stomata to open.

The highly permeable cuticle in young, expanding leaves previously observed in *Quercus macrocarpa*, *Q. muehlenbergii*, and *H. helix* (Hamerlynck and Knapp, 1996; Hauke and Schreiber, 1998) may be due to the development of the cuticle (Lee and Priestley, 1924; Neinhuis et al., 2001). Mature cuticles are extremely dense with a very high breakage strength, suggesting that a weaker cuticle may be necessary to allow cells and leaves to expand (Onoda et al., 2012). The more elastic disjointed developing cuticle needed to allow cell expansion may come at the cost of a higher cuticular conductance. If this is the case, plants would have to balance the maintenance of high turgor pressure to drive cell expansion and deliver nutrients with a permeable cuticle to allow for cell expansion. Although cuticle permeance has been found to be a function of water status with high leaf water potential leading to higher levels of cuticular water loss (Boyer et al., 1997; Jordan and Brodribb, 2007), it is unlikely that the high levels of cuticular water loss in young leaves might simply be due to the higher water status of young expanding leaves as these leaves have the same water potentials as fully expanded leaves. This is in agreement with previous work in other *Quercus* species, in which there was no difference found in leaf water potential across leaf age as leaves expand (Ren and Sucoff, 1995; Hamerlynck and Knapp, 1996). In *Q. rubra* we observed much thinner cuticles in younger leaves when compared to those that were fully expanded; this anatomical change in cuticle thickness and possibly composition is the likely cause of the higher cuticular water loss measured in young expanding leaves.

Our work suggests that the formation of the outer cuticular ledge above stomata of developing leaves (and therefore formation of an aperture) could be a major determinant of the timing and relevance of stomatal function in leaf gas exchange. Here, we observed that stomatal water loss only occurs when stomata have these apertures (Figures 1, 4). The cuticle that covers stomata before the formation of the outer cuticular ledge likely inhibits water flux through individual stomatal pores, just as it reduces stomatal conductance in *A. thaliana* mutant plants that do not form an outer cuticular ledge (Hunt et al., 2017). Once that cuticle tears and the outer cuticular ledge is formed, *Q. rubra* stomata are capable of sustaining maximum water loss rates through the pore. These cuticle coverings in young stomata have been observed multiple times in *A. thaliana* (Serna and Fenoll, 1997; Nadeau and Sack, 2002; Hunt et al., 2017), in *Hydrocotyle bonariensis* (Koch and Barthlott, 2009), the stomata on the flowers of *Vicia faba* (Davis and Gunning, 1993), and now *Q. rubra*. Given that we observed these in both *Q. rubra* and *A. thaliana*, and stomatal development and developmental genes are highly conserved across land plants, this cuticular covering of young stomata may be a feature common to all vascular plants (Chater et al., 2017). Whether it extends to non-vascular plant stomata remains to be examined (Renzaglia et al., 2017).

The extremely high levels of ABA found in young leaves of *Q. rubra* could have several explanations all requiring future examination. It is possible that the newest expanding leaves have high levels of ABA because ABA is required to maintain bud dormancy (Kovaleski and Londo, 2019). The decreases seen here as leaves expand might be due to dilution and catabolism as bud dormancy is broken (Kovaleski and Londo, 2019). The ABA may also be playing a role in cuticle formation, as some ABA deficient tomato mutants have thinner cuticles with reduced levels of cutin that are partially restored by the application of ABA (Martin et al., 2017). Another possibility is that ABA may be responsible for maintaining low guard cell turgor during leaf development to stop the premature tearing of the cuticle covering above the stomatal pore. Exogenous applications of ABA have been found to keep stomata closed under the cuticle covering in *focl* mutants, which have much reduced formation of the outer cuticular ledge, indicating that stomata that have a cuticle covering are possibly capable of opening and closing (Hunt et al., 2017). There is the possibility that the high levels of ABA in young leaves may be sequestered in chloroplasts, and this fettered ABA is non-functional (Loveys, 1977; Georgopoulou and Milborrow, 2012). However, given the observation in an evergreen *Quercus* species and other herbaceous species that chloroplast number is very low in young, expanding leaves, increasing as leaves expand (Miyazawa et al., 2003), this possibility seems unlikely. The most likely explanation is that the high levels of ABA found in the expanding leaves of *Q. rubra* are responsible for keeping stomata closed as leaves expand; although given other signals can close stomata (Granot et al., 2013; Salmon et al., 2020), more experimental work is required to test this theory.

Based on this work, the apparent order of events in expanding *Q. rubra* leaves is that very young leaves have relatively high levels of cuticular water loss that decline as leaves cease expanding. During expansion, stomata develop, but are present in low numbers and covered with a cuticle. Foliage ABA levels are initially high and decrease through time as leaves expand, possibly keeping the youngest stomata closed under the cuticle, until the cuticle connecting the guard cells tears to form the stomatal aperture, or is torn open by the opening stomata. Once the outer cuticular ledge forms, stomata account for most of the water lost from expanded leaves. This chain of events is very different to the model proposed by Pantin et al. (2013) based on observations made in *A. thaliana*. We would argue that these differences are not due to differences in species, as we found similar morphological development in the expanding leaves of both *Quercus* and *Arabidopsis*. However, further work is required to investigate the importance of cuticular conductance in leaf gas exchange as leaves expand across a wide diversity of species and also under field conditions. We find that the model of Pantin et al. (2013) is not supported by our observations of very high levels of ABA measured in young leaves, the cuticle covering of young stomata, and the relatively late development of the outer cuticular ledge in expanding leaves of *A. thaliana* and *Q. rubra*, all of which run counter to the theory that stomata are wide open and responsible for all of the water loss from young, expanding leaves. We conclude that the cuticle plays a primary role in determining the rate of water loss from expanding leaves.

## Data Availability Statement

The datasets generated for this study are available on request to the corresponding author.

## Author Contributions

This work was originally conceived by SM with GJ. SJ assisted with collection of SEM images and preparation of anatomical samples. All data was collected and analyzed by CK under the supervision of SM. Manuscript was written by CK with input from SM, GJ, and SJ. All authors contributed to the article and approved the submitted version.

### Conflict of Interest

The authors declare that the research was conducted in the absence of any commercial or financial relationships that could be construed as a potential conflict of interest.

## AcknowleDgments

We acknowledge the use of the facilities of the Bindley Bioscience Center (National Institutes of Health-funded Indiana Clinical and Translational Sciences Institute), particularly the Metabolite Profiling Facility. We would also like to thank Robert Seiler at the Purdue Life Science Microscopy Facility for help with the cryoSEM, Dr. Jennifer McElwain for a helpful discussion on stomatal development, and Justine Krueger for collecting insightful preliminary data that led to this study.

## References

[ref1] Blackman C. J. Pfautsch S. Choat B. Delzon S. Gleason S. M. Duursma R. A. (2016). Toward an index of desiccation time to tree mortality under drought. Plant Cell Environ. 39, 2342–2345. 10.1111/pce.12758, PMID: 27093688

[ref2] Bolhàr-Nordenkampf H. R. Draxler G. (1993). “Functional leaf anatomy” in Photosynthesis and production in a changing environment: A field and laboratory manual. eds. Hall D. O. Scurlock J. M. O. Bolhàr-Nordenkampf H. R. Leegood R. C. Long S. P. (Netherlands: Springer), 91–112.

[ref3] Boyer J. S. Wong S. C. Farquhar G. D. (1997). CO_2_ and water vapor exchange across leaf cuticle (epidermis) at various water potentials. Plant Physiol. 114, 185–191. 10.1104/pp.114.1.185, PMID: 12223698 PMC158293

[ref4] Brodribb T. J. McAdam S. A. M. Jordan G. J. Martins S. C. V. (2014). Conifer species adapt to low-rainfall climates by following one of two divergent pathways. Proc. Natl. Acad. Sci. U. S. A. 111, 14489–14493. 10.1073/pnas.1407930111, PMID: 25246559 PMC4210017

[ref5] Brodribb T. J. Sussmilch F. McAdam S. A. M. (2020). From reproduction to production, stomata are the master regulators. Plant J. 101, 756–767. 10.1111/tpj.14561, PMID: 31596990

[ref6] Buckley T. N. Mott K. A. Farquhar G. D. (2003). A hydromechanical and biochemical model of stomatal conductance. Plant Cell Environ. 26, 1767–1785. 10.1046/j.1365-3040.2003.01094.x

[ref63] Burkhardt J. Pariyar S. (2014). Particulate pollutants are capable to ‘degrade’ epicuticular waxes and to decrease the drought tolerance of Scots Pine (Pinus sylvestris L.). Environ. Pollution 184, 659–667. 10.1016/j.envpol.2013.04.041 23791043

[ref7] Buschhaus C. Herz H. Jetter R. (2007). Chemical composition of the epicuticular and intracuticular wax layers on adaxial sides of *Rosa canina* leaves. Ann. Bot. 100, 1557–1564. 10.1093/aob/mcm255, PMID: 17933845 PMC2759234

[ref64] Carignato A. Vázquez-Piqué J. Tapias R. Ruiz F. Fernández M. (2020) Variability and plasticity in cuticular transpiration and leaf permeability allow differentiation of Eucalyptus clones at an early age. Forests 11:9.

[ref8] Chater C. C. C. Caine R. S. Fleming A. J. Gray J. E. (2017). Origins and evolution of stomatal development. Plant Physiol. 174, 624–638. 10.1104/pp.17.00183, PMID: 28356502 PMC5462063

[ref9] Constable G. A. Rawson H. M. (1980). Effect of leaf position, expansion and age on photosynthesis, transpiration and water use efficiency of cotton. Funct. Plant Biol. 7, 89–100. 10.1071/PP9800089

[ref10] Davis A. R. Gunning B. E. S. (1993). The modified stomata of the floral nectary of *Vicia faba* L. 1. Development, anatomy and ultrastructure. Plant Biol. 106, 241–253. 10.1111/j.1438-8677.1993.tb00747.x

[ref11] Duursma R. A. Blackman C. J. Lopéz R. Martin-StPaul N. K. Cochard H. Medlyn B. E. (2019). On the minimum leaf conductance: its role in models of plant water use, and ecological and environmental controls. New Phytol. 221, 693–705. 10.1111/nph.15395 30144393

[ref12] Edwards D. Abbott G. D. Raven J. A. (1996). “Cuticles of early land plants: a palaeoecophysiological evaluation” in Plant cuticles an integrated functional approach. ed. Kerstiens G. (Oxford: BIOS Scientific Publishers), 1–31.

[ref13] Fernández V. Guzmán-Delgado P. Graça J. Santos S. Gil L. (2016). Cuticle structure in relation to chemical composition: re-assessing the prevailing model. Front. Plant Sci. 7:427. 10.3389/fpls.2016.00427, PMID: 27066059 PMC4814898

[ref14] Georgopoulou Z. Milborrow B. V. (2012). Initiation of the synthesis of ‘stress’ ABA by (+)-[^2^H_6_]ABA infiltrated into leaves of *Commelina communis*. Physiol. Plant. 146, 149–159. 10.1111/j.1399-3054.2012.01630.x, PMID: 22471592

[ref15] Goodwin S. M. Jenks M. A. (2005). “Plant cuticle function as a barrier to water loss” in Plant abiotic stress. eds. Jenks M. A. Hasegawa P. M. (Oxford: Blackwell Publishing), 14–31.

[ref16] Granot D. Kelly G. Stein O. David-Schwartz R. (2013). Substantial roles of hexokinase and fructokinase in the effects of sugars on plant physiology and development. J. Exp. Bot. 65, 809–819. 10.1093/jxb/ert400 24293612

[ref17] Gülz P.-G. (1994). Epicuticular leaf waxes in the evolution of the plant kingdom. J. Plant Physiol. 143, 453–464. 10.1016/S0176-1617(11)81807-9

[ref18] Hamerlynck E. P. Knapp A. K. (1996). Early season cuticular conductance and gas exchange in two oaks near the western edge of their range. Trees 10, 403–409. 10.1007/BF02185644

[ref19] Hauke V. Schreiber L. (1998). Ontogenetic and seasonal development of wax composition and cuticular transpiration of ivy (*Hedera helix* L.) sun and shade leaves. Planta 207, 67–75. 10.1007/s004250050456

[ref20] Hsiao T. C. Xu L.-K. (2000). Sensitivity of growth of roots versus leaves to water stress: biophysical analysis and relation to water transport. J. Exp. Bot. 51, 1595–1616. 10.1093/jexbot/51.350.1595, PMID: 11006310

[ref21] Hunt L. Amsbury S. Baillie A. Movahedi M. Mitchell A. Afsharinafar M.. (2017). Formation of the stomatal outer cuticular ledge requires a guard cell wall proline-rich protein. Plant Physiol. 174, 689–699. 10.1104/pp.16.01715, PMID: 28153922 PMC5462008

[ref22] Ivănescu L. Lăzărescu A. M. Toma C. (2009). Comparative anatomy of the foliar lamina in some taxa of *Quercus* L. genus. *Analele Ştiinţifice Ale Universităţii “Al. I. Cuza” Iaşi Tomul LV, Fasc. 2, s.II a. Biologie Vegetală*, 7.

[ref23] Jeffree C. E. (1996). “Structure and ontogeny of plant cuticles” in Plant cuticles an integrated functional approach. ed. Kerstiens G. (Oxford: BIOS Scientific Publishers), 33–82.

[ref24] Jordan G. J. Brodribb T. J. (2007). Incontinence in aging leaves: deteriorating water relations with leaf age in *Agastachys odorata* (proteaceae), a shrub with very long-lived leaves. Funct. Plant Biol. 34, 918–924. 10.1071/FP07166 32689420

[ref25] Kenrick P. Crane P. R. (1997). The origin and early evolution of plants on land. Nature 389, 33–39. 10.1038/37918

[ref26] Koch K. Barthlott W. (2009). Superhydrophobic and superhydrophilic plant surfaces: an inspiration for biomimetic materials. Philos. Trans. R Soc. A Math. Phys. Eng. Sci. 367, 1487–1509. 10.1098/rsta.2009.0022, PMID: 19324720

[ref65] Körner C. Scheel J. A. Bauer A. (1979). Maximum leaf diffusive conductance in vascular plants. Photosynthetica 13, 45–82.

[ref27] Körner C. (1993). “Scaling from species to vegetation: the usefulness of functional groups” in Biodiversity and ecosystem function. eds. Schulze E.-D. Mooney H. A. (Berlin Heidelberg: Springer), 117–140.

[ref28] Kovaleski A. P. Londo J. P. (2019). Tempo of gene regulation in wild and cultivated *Vitis* species shows coordination between cold deacclimation and budbreak. Plant Sci. 287:110178. 10.1016/j.plantsci.2019.110178, PMID: 31481199

[ref29] Krauss P. Markstädter C. Riederer M. (1997). Attenuation of UV radiation by plant cuticles from woody species. Plant Cell Environ. 20, 1079–1085. 10.1111/j.1365-3040.1997.tb00684.x

[ref30] Łaźniewska J. Macioszek V. K. Kononowicz A. K. (2012). Plant-fungus interface: the role of surface structures in plant resistance and susceptibility to pathogenic fungi. Physiol. Mol. Plant Pathol. 78, 24–30. 10.1016/j.pmpp.2012.01.004

[ref31] Lee B. Priestley J. H. (1924). The plant cuticle I its structure, distribution, and function. Ann. Bot. os-38, 525–545. 10.1093/oxfordjournals.aob.a089915

[ref32] Lee S. B. Yang S. U. Pandey G. Kim M.-S. Hyoung S. Choi D. (2019). Occurrence of land-plant-specific glycerol-3-phosphate acyltransferases is essential for cuticle formation and gametophore development in *Physcomitrella patens*. New Phytol. 225, 2468–2483. 10.1111/nph.16311 31691980

[ref33] Lendzian K. J. (1982). Gas permeability of plant cuticles: oxygen permeability. Planta 155, 310–315. 10.1007/BF00429457, PMID: 24271865

[ref34] Lendzian K. J. Kerstiens G. (1991). “Sorption and transport of gases and vapors in plant cuticles” in Reviews of environmental contamination and toxicology: Continuation of residue reviews. ed. Ware G. W. (New York: Springer), 65–128.

[ref35] Liu F. Jensen C. R. Andersen M. N. (2003). Hydraulic and chemical signals in the control of leaf expansion and stomatal conductance in soybean exposed to drought stress. Funct. Plant Biol. 30, 65–73. 10.1071/FP02170 32688993

[ref36] Loveys B. R. (1977). The intracellular location of abscisic acid in stressed and non-stressed leaf tissue. Physiol. Plant. 40, 6–10. 10.1111/j.1399-3054.1977.tb01483.x

[ref37] Martin L. B. B. Romero P. Fich E. A. Domozych D. S. Rose J. K. C. (2017). Cuticle biosynthesis in tomato leaves is developmentally regulated by abscisic acid. Plant Physiol. 174, 1384–1398. 10.1104/pp.17.00387, PMID: 28483881 PMC5490907

[ref38] Mayr S. (2007). “Limits in water relations” in Trees at their upper limit: Treelife limitation at the alpine timberline. eds. Wieser G. Tausz M. (Netherlands: Springer), 145–162.

[ref39] McAdam S. (2015). Physicochemical quantification of abscisic acid levels in plant tissues with an added internal standard by ultra-performance liquid chromatography. Bio. Protoc. 5:e1599. 10.21769/BioProtoc.1599

[ref40] Medeiros C. D. Falcão H. M. Almeida-Cortez J. Santos D. Y. A. C. Oliveira A. F. M. Santos M. G. (2017). Leaf epicuticular wax content changes under different rainfall regimes, and its removal affects the leaf chlorophyll content and gas exchanges of *Aspidosperma pyrifolium* in a seasonally dry tropical forest. S. Afr. J. Bot. 111, 267–274. 10.1016/j.sajb.2017.03.033

[ref41] Miyazawa S.-I. Makino A. Terashima I. (2003). Changes in mesophyll anatomy and sink-source relationships during leaf development in *Quercus glauca*, an evergreen tree showing delayed leaf greening. Plant Cell Environ. 26, 745–755. 10.1046/j.1365-3040.2003.01011.x

[ref42] Nadeau J. A. Sack F. D. (2002). Stomatal development in arabidopsis. Arabidopsis Book 1:e0066. 10.1199/tab.0066, PMID: 22303215 PMC3243354

[ref43] Neinhuis C. Koch K. Barthlott W. (2001). Movement and regeneration of epicuticular waxes through plant cuticles. Planta 213, 427–434. 10.1007/s004250100530, PMID: 11506366

[ref44] Onoda Y. Richards L. Westoby M. (2012). The importance of leaf cuticle for carbon economy and mechanical strength. New Phytol. 196, 441–447. 10.1111/j.1469-8137.2012.04263.x, PMID: 22913608

[ref45] Pantin F. Renaud J. Barbier F. Vavasseur A. Le Thiec D. Rose C.. (2013). Developmental priming of stomatal sensitivity to abscisic acid by leaf microclimate. Curr. Biol. 23, 1805–1811. 10.1016/j.cub.2013.07.050, PMID: 24035546

[ref46] Pantin F. Simonneau T. Muller B. (2012). Coming of leaf age: control of growth by hydraulics and metabolics during leaf ontogeny. New Phytol. 196, 349–366. 10.1111/j.1469-8137.2012.04273.x, PMID: 22924516

[ref47] Pereira S. Figueiredo-Lima K. Oliveira A. F. M. Santos M. G. (2019). Changes in foliar epicuticular wax and photosynthesis metabolism in evergreen woody species under different soil water availability. Photosynthetica 57, 192–201. 10.32615/ps.2019.013

[ref48] Raven J. A. (1984). Physiological correlates of the morphology of early vascular plants. Bot. J. Linn. Soc. 88, 105–126. 10.1111/j.1095-8339.1984.tb01566.x

[ref49] Ren Z. Sucoff E. (1995). Water movement through *Quercus rubra* I. leaf water potential and conductance during polycyclic growth. Plant Cell Environ. 18, 447–453. 10.1111/j.1365-3040.1995.tb00379.x

[ref50] Renzaglia K. S. Villarreal J. C. Piatkowski B. T. Lucas J. R. Merced A. (2017). Hornwort stomata: architecture and fate shared with 400-million-year-old fossil plants without leaves. Plant Physiol. 174, 788–797. 10.1104/pp.17.00156, PMID: 28584065 PMC5462037

[ref51] Salmon Y. Lintunen A. Dayet A. Chan T. Dewar R. Vesala T.. (2020). Leaf carbon and water status control stomatal and nonstomatal limitations of photosynthesis in trees. New Phytol. 226, 690–703. 10.1111/nph.16436, PMID: 31955422

[ref52] Sansberro P. A. Mroginski L. A. Bottini R. (2004). Foliar sprays with ABA promote growth of *Ilex paraguariensis* by alleviating diurnal water stress. Plant Growth Regul. 42, 105–111. 10.1023/B:GROW.0000017476.12491.02

[ref53] Šantrůček J. Šimáňová E. Karbulková J. Šimková M. Schreiber L. (2004). A new technique for measurement of water permeability of stomatous cuticular membranes isolated from *Hedera helix* leaves. J. Exp. Bot. 55, 1411–1422. 10.1093/jxb/erh150, PMID: 15155780

[ref54] Sargent C. (1976). The occurrence of a secondary cuticle in *Libertia elegans* (Iridaceae). Ann. Bot. 40, 355–359. 10.1093/oxfordjournals.aob.a085138

[ref55] Schreiber L. (2005). Polar paths of diffusion across plant cuticles: new evidence for an old hypothesis. Ann. Bot. 95, 1069–1073. 10.1093/aob/mci122, PMID: 15797897 PMC4246894

[ref56] Schreiber L. Riederer M. (1996). Ecophysiology of cuticular transpiration: comparative investigation of cuticular water permeability of plant species from different habitats. Oecologia 107, 426–432. 10.1007/BF00333931, PMID: 28307383

[ref57] Schultz H. R. Matthews M. A. (1993). Growth, osmotic adjustment, and cell-wall mechanics of expanding grape leaves during water deficits. Crop Sci. 33, 287–294. 10.2135/cropsci1993.0011183X003300020015x

[ref58] Serna L. Fenoll C. (1997). Tracing the ontogeny of stomatal clusters in arabidopsis with molecular markers. Plant J. 12, 747–755. 10.1046/j.1365-313X.1997.12040747.x, PMID: 9375390

[ref59] Shackel K. Matthewes M. Morrison J. (1987). Dynamic relation between expansion and cellular turgor in growing grape (*Vitis vinifera* L.) leaves. Plant Physiol. 84, 1166–1171. 10.1104/pp.84.4.1166, PMID: 16665579 PMC1056746

[ref60] Siebrecht S. Herdel K. Schurr U. Tischner R. (2003). Nutrient translocation in the xylem of poplar—diurnal variations and spatial distribution along the shoot axis. Planta 217, 783–793. 10.1007/s00425-003-1041-4, PMID: 12721678

[ref61] Tomlinson P. T. Dickson R. E. Isebrands J. G. (1991). Acropetal leaf differentiation in *Quercus rubra* (*Fagaceae*). Am. J. Bot. 78, 1570–1575. 10.1002/j.1537-2197.1991.tb11436.x

[ref62] Yeats T. H. Rose J. K. C. (2013). The formation and function of plant cuticles. Plant Physiol. 163, 5–20. 10.1104/pp.113.222737, PMID: 23893170 PMC3762664

